# Beta estimation in the European network regulation context: what matters, what doesn’t, and what is indispensable

**DOI:** 10.1007/s11408-023-00428-z

**Published:** 2023-04-26

**Authors:** Dmitry Bazhutov, André Betzer, Richard Stehle

**Affiliations:** 1 Schumpeter School of Business and Economics, University of Wuppertal, Gaußstr. 20, 42119 Wuppertal, Germany; 2 School of Business and Economics, Humboldt University Berlin, Spandauer Str. 1, 10178 Berlin, Germany

**Keywords:** Cost of capital, Beta, Coronavirus, Regulated firms, C52, G12, L94, L95, L96

## Abstract

Most studies on beta estimation look at the whole universe of stocks. We focus on a small subset that consists of stocks of companies which are subject to European network regulation. This allows us to examine beta time series of individual stocks and small peer groups in great detail. Our most important conclusions are: (1) Sudden beta increases or decreases occur that often last only short periods of time and may therefore cause a significant misestimation of the future beta. (2) Three- and especially five-year betas are much more stable than one-year betas. (3) The choice between purely local, European or global betas may matter considerably. (4) Weekly or daily betas seem to be better than monthly ones. (5) Vasicek and Blume adjustments towards one lead to beta predictions that are too high.

## Introduction and summary

In Europe—like in many other countries—firms that have a natural monopoly are regulated to prevent them from charging prices that deviate too much from the prices under perfect competition. The main subjects of this regulation are the operators of gas, electricity, and telecommunication networks as well as water suppliers, airport operators, and operators of railroad infrastructures. Since these are capital intensive industries, the cost of capital plays an important part in their regulation. National regulators undertake extensive consulting procedures involving all concerned parties to determine the allowed cost of capital that may be used in price setting. UKRN (2020) describes the current procedures used to estimate the cost of capital of network operators in the UK in all areas of regulation; Stehle (2016) describes the procedures used in the German telecommunications sector. At this point the most recent regulatory publication is Frontier (2021).

Since the cost of equity capital cannot be observed, neither ex ante nor ex post, a model must be used to help estimate the equity cost of capital. For many years, especially in Europe, the capital asset pricing model (CAPM) developed by Sharpe (1964) and Lintner (1965) has been used for this purpose. Ideally, three direct inputs are needed to implement this model: the “true” risk-free rate of interest, the “true” market risk premium and the “true” beta, which is a measure of the non-diversifiable risk of the network operator’s stocks. These three values cannot be observed, but only estimated. This paper focuses on beta estimation. More precisely, on the estimation of the equity betas. Typically, the equity betas are an input in the estimation of the asset betas, which in turn are used in the calculation of the “relevered betas”.^1^

In network regulation, equity betas are almost always estimated for a sample of firms, that is for a peer group. Using a peer group is the only way to estimate the beta of an unlisted firm and it provides, if properly constructed, a better estimate of the beta of a listed company. Ideally the peer group should consist of pure play firms whose shares are traded in the capital market in which the regulation takes place. In our context pure play firms are firms which only have activities in the area on which the regulatory efforts focus and which operate under the same regulatory regime.

Beta estimates are subject to estimation errors and risk instability. A number of academic studies address these issues (e.g., Hollstein 2020; Hollstein et al. 2019; Levi and Welch 2017; Draper and Paudyal 1995). These and most other studies on beta estimation look at the whole universe of stocks. We concentrate on the small subset of European network operators. This allows us to closely examine beta time series of individual stocks whose operations we know well and of small peer groups consisting of firms that come close to the ideal of pure plays.

In the area of network regulation and likely in many other contexts, it makes little sense to use the results for the universe of all betas, since the market-value weighted average of all betas, calculated in an identical way, always equals one on every single day in history, even in a financial crisis. In line with that, prior studies document the heterogenous effects of the 2008 financial crisis on the systematic risk of individual firms which has increased for some and decreased for other firms (e.g., Ben Slimane et al. 2017; Grout and Zalewska 2016). We are only interested in how economic shocks affect the betas of firms subject to European network regulation.

Network regulation was traditionally based on national laws and enforced by national regulators. An important economic objective of the EU is to create a single internal market comprising all member countries with free movements of goods, capital, services and labor. An important instrument in this context is EU Directives which member countries must translate into their national legislation within a defined time frame. In the different areas of network regulation, especially electricity, gas and telecommunication such regulations have started at the beginning of this century and have become more detailed in the meantime (e.g., directives 2003/55/EC, 2009/73/EC, and 2019/692 for the gas market).

Regulated firms are typically small or medium-sized companies which have relatively stable betas (often significantly) below one. Small beta differences may matter a lot to the parties involved: directly involved parties include the firm’s investors, managers, and customers; indirectly a country’s entire population may be involved. Focusing on a small number of individual stocks and on peer groups consisting of these stocks allows us to analyze ten-year beta time series (2010–2020) in which the beta estimates are calculated in different ways. Like nearly all prior studies, we use ordinary least squares (OLS) to estimate betas and use only one independent variable, a stock market index. Despite these simplifications, many important details have to be set in the estimation process, which we discuss.

We consider it self-evident, (1) that we should use total returns (returns that include dividends), and (2) that all returns should be expressed in the same currency, we always use euros. We also think it is obvious that we have to start out by cleaning the data, then calculate betas, and finally look at the adjustments suggested by Vasicek (1973) and Blume (1971, 1975).

In Sect. 2, we present our data sources and cleaning procedures. In our main Sect. 3, we present and interpret the graphs of beta time series calculated in several different ways, such as using:

- Simple returns, excess returns or logarithmic returns
- Alternative indices: local indices, the EU-wide Stoxx 600, and the worldwide MSCI
- One-year, three-year, and five-year observation windows, i.e. the length of the time period on which the estimate is based
- Daily, weekly, and monthly return intervals, i.e. the type of returns used in OLS
- Individual stocks and peer groups

In most cases, we can draw a firm conclusion from a visual interpretation of the graphed data. In some instances, a statistical analysis further strengthens our findings.

In Sect. 3.1, we look at the use of (simple) returns, logarithmic returns, and excess returns (returns minus the risk-free rate) and assess whether applying one or the other makes a difference. We find that it does not matter.

In Sect. 3.2, we focus on the choice of an index. Often different indices produce very similar betas, but for specific time periods and firms the index choice may matter considerably. We also discuss theoretical arguments for using an international index when the firms in a peer group reside in different countries.

In Sect. 3.3, we start to discuss the observation windows. We use several graphs that clearly show that one-year betas (betas calculated with returns in the prior year) fluctuate strongly and randomly around their long-term mean and therefore are not useful in the regulatory context. Their standard deviation is typically twice as large as that of three- and five-year betas, while long-term means are very similar. In the regulation context, the beta during the regulation period has to be predicted one to two years before the start of this period. Because of the seemingly random variation over time, the most recent one-year beta is not very useful. We also would not recommend to include it in an average of betas based on different observation windows.^2^ Five-year betas are more stable than three-year betas and have other desirable properties in our context. This is why we recommend them.

In Sect. 3.4, we cover the return interval. Monthly betas—betas based on monthly rates of return—often differ significantly from both daily and weekly betas, which are roughly similar. Weekly returns are superior to daily data in the case of less liquid stocks. We also note that rates of return may be based on different trading hours because the stock exchanges are located in different countries. This affects daily betas more than weekly betas. For these reasons, we recommend weekly betas for peer groups that are international and/or include smaller firms. We recommend daily betas if the whole peer group consists of larger companies with main listings on the same exchange.

In our sample, three- and five-year betas typically increase or decrease slowly over time. But we notice sudden and sharp rises and falls directly or indirectly caused by the 2008 worldwide economic crisis and the recent 2020 coronavirus pandemic. In Sect. 3.5, we focus on these sudden beta increases and decreases. The sudden beta increases or decreases that are indirectly caused by the economic shocks show up several years after the crisis and are a consequence of the calculation procedure.

Since most of these sudden increases and decreases at least partially reverse within short time periods, the most important conclusion of our paper is that looking at the beta of a single date is not sufficient in the regulatory context. It is indispensable to look at beta time series for several years or at least at several beta estimates for different points in time. If this is not possible, it may be helpful to look at the beta estimates of prior studies to avoid errors. Similarly, we find it indispensable to use a peer group to estimate the beta.

Three- and five-year betas are fairly stable over time, and for some firms and peer groups very stable over time. When we see a beta increase over time, we often observe (ex post) that the firm added new or international activities to its portfolio. These possibly lead to higher betas in the long run—higher than the beta in the years in which the firm concentrated on regulated activities. In Sect. 4, we argue that because of the stability of the regulated activities, Blume or Vasicek adjustments towards one lead to betas which are too high.

Overall, we recommend starting out by cleaning the data, calculating raw betas, omitting adjustments and using five-year betas based on weekly data. We recommend thoroughly screening the firms that may form a peer group for activities outside of the regulated activities in their home country. Once the firms of the peer group are chosen carefully, a small peer group may be sufficient. The British Water Services Regulation Authority, or Ofwat, followed this procedure recently for the upcoming regulatory period. They excluded one British water company because a significant portion of its revenues are derived from activities outside of British water regulation and formed a peer group of only two purely water companies (Ofwat 2019, p. 54).

Our results are of particular interest for practitioners involved in the estimation of the cost of equity capital. Whatever the context, it is necessary to look not only at the current beta but at the past beta time series to avoid biased point estimates. Our results should also be taken into account in future academic studies that investigate the universe of all stocks because it is not easy to identify the sudden beta increases and decreases when looking at the whole universe of stocks and so far they have been overlooked, e.g., Levi and Welch (2017), Hollstein (2020). Last but not least we also contribute to the comprehensive regulatory literature (e.g., Robertson 2018; Indepen 2018; Henry 2014) by providing important implications for the future beta estimation process. We notice that the significant beta increase during the recent coronavirus pandemic in 2020 may affect the betas of regulated firms for several years—and especially at the time when the related observations fall out of the sample. Future studies should be aware of this issue.

## Sample, data, and data cleaning

In our study, we rely on a sample of twelve European firms from the utility and telecommunication sectors in order to visualize several details of beta estimation and to identify different issues related to it. There are three main reasons for analyzing these firms in our context. First, the activities of our sample firms are regulated by government agencies, making the detailed process of beta estimation for this group of firms of particular interest to all parties involved: regulators, network operators, consulting firms, and academics. Second, firms conducting regulated activities are frequently assumed to be less affected by economic and market fluctuations compared with most other firms, so investigating them should allow us to better highlight the "technical" effects of beta estimation. Finally, during the recent economic shock resulting from the coronavirus pandemic, the aforementioned assumptions regarding regulated firms seem to no longer hold true. For instance, the Wall Street Journal (2020) reports that "Safe Utilities Have Been More Volatile Than Broader Stock Market" and "[t]he dynamic we saw this year with COVID-19 is something that no one knew how to model (…)".^3^ We aim to address this issue in detail using the evidence from our sample.

Our sample consists of three pure electricity network operators, two pure gas network operators, and two operators of both electricity and gas networks, three major telecommunication network operators, and finally two pure water companies*.* By pure we mean that the companies had at least 90% of their activities in the given area. We do not include two companies in our sample due to lack of sufficient data.^4^ Reasons to exclude firms might be different, for instance, the required stock price data may become available only during the sample period (e.g., after an IPO) or it is no longer available towards the end of the period (e.g., because of a takeover). In addition, stock returns could be disproportionately volatile, for instance, when there is no sufficient free float of the stocks. Since all of the above issues might impede consistent estimation, we make sure that they do not apply to our sample. An overview of the sample firms is provided in Table 1.

**Table 1:** Overview of sample firms

Firm	Country	National index
*Electricity network operators*		
Elia Group SA/NV	Belgium	BEL 20
Red Electrica Corporacion S.A	Spain	IBEX 35
Terna—Rete Elettrica Nazionale S.p.A	Italy	FTSE MIB
*Gas network operators*		
Enagás, S.A	Spain	IBEX 35
Snam S.p.A	Italy	FTSE MIB
*Electricity and gas network operators*		
Redes Energeticas Nacionais SGPS S.A	Portugal	PSI 20
National Grid plc	United Kingdom	FTSE 100
*Telecommunication network operators*		
Deutsche Telekom AG	Germany	DAX 30
Vodafone Group plc	United Kingdom	FTSE 100
Telefónica S.A	Spain	IBEX 35
*Water utilities*		
Severn Trent plc	United Kingdom	FTSE 100
United Utilities Group plc	United Kingdom	FTSE 100

This table provides an overview of twelve European firms in our sample by industry sectors. The headquarter countries and the corresponding national stock indices are reported accordingly. We use the total return version of all national stock indices (i.e., including dividends) in all our analyses

In addition, to calculate the market returns required for beta estimation, we use different national, European, and worldwide stock indices. We particularly rely on the Stoxx Europe 600 as a broad European index and the MSCI World as a worldwide index. Table 1 also shows the major national stock market indices related to each firm in our study.

All market data for our sample is obtained from the Refinitiv Datastream database. To calculate the returns of stocks and indices, we use total return indices (abbr. "RI" in Datastream) instead of raw prices. That is because the total returns of a stock or an index include—in addition to the price change—other relevant return components, in particular, dividend payments.^5^ Total returns also account for corporate actions, such as stock splits. The latter can cause serious estimation errors when using price changes only, since a stock split has only a technical effect on the stock price and does not affect overall return or beta.^6^ While some stock market indices are calculated as total return indices by default some others do not include dividend payments. We consistently use total returns with all stocks and indices that are calculated using the same methodology.^7^

Furthermore, when betas are estimated for a sample of international firms and/or when an international or global index is used, it should be made sure that a beta of a firm is estimated from stock and index returns that are denominated in the same currency. Because most of the firms in our sample belong to the euro area, we use the euro as our primary currency. For consistency, we convert the returns of the British firms in our sample to euro returns and use the FTSE 100 index and the MSCI World index denominated in euros.

As we rely on international stock indices—in addition to local ones—to calculate market returns, national holidays may become an issue, because country specific national holidays are associated with the absence of local exchange trading, but the international market index is still calculated. For instance, if a national holiday falls on Friday, a weekly stock return would cover four trading days, while the return of the international stock index would include five days. Furthermore, the stock return of the next week would correspond to a six-day market return. This issue is even more pronounced with daily data, as most commercial databases pad non-trading days with the last price values. So a number of zero stock returns can result when daily data is used and, as a consequence, betas could be underestimated.

A common technique for dealing with problems related to non-trading days is the trade-to-trade approach suggested by Schwert (1977) and Franks et al. (1977). With this technique, the stock returns are calculated only between trading days and the corresponding market returns are computed for exactly the same time interval, so that both return series fit together.^8^ Since there are several national non-trading days per year associated with our sample, we use trade-to-trade returns in all of our estimations and organize our data accordingly. We do not apply the adjustment suggested by Marsh (1979), which aims to address the heteroscedasticity issues associated with the trade-to-trade approach, as this problem seems to be negligible in our context.

After preparing and cleaning the data, betas can be calculated. This we discuss in the next section.

## Nuances of beta estimation: what matters and what doesn’t

### Return calculation

The “true” beta we are looking for in the regulatory context is the beta in the future regulatory period, which, according to the CAPM, is the future value of $C\!O\!V{(R}_{i},{R}_{m})/\mathrm{VAR}({R}_{m})$, which measures the sensitivity of stock returns with respect to market returns. Beta is usually estimated by using the market model (or single-index model) suggested by Sharpe (1963) and the OLS technique. The market model equation is:

1 $$R_{i,t} = \alpha_{i} + \beta_{i} R_{m,t} + \varepsilon_{i,t}$$

where:

${R}_{i,t}$= the return of a stock $i$ in period $t$

${R}_{m,t}$= the return of a market portfolio $m$ in period 

${\alpha }_{i}$ = the constant term

${\varepsilon }_{i,t}$ = the homoscedastic error term

${\beta }_{i}$ = the equity beta of a stock $i$

Given homoscedasticity, a regular assumption in most studies, OLS provides the best unbiased beta estimate ${\widehat{\beta }}_{i}$.

From a theoretical perspective, when estimating the cost of equity using the CAPM, the betas should be calculated using excess returns. Excess returns are obtained by subtracting the risk-free rate (${R}_{f,t}$, which typically equals the return of a short-term treasury bill) from stock and market returns, yielding the following equation:

2 $$R_{i,t} - R_{f,t} = \alpha_{i} + \beta_{i} (R_{m,t} - R_{f,t} ) + \varepsilon_{i,t}$$

**Fig. 1:**
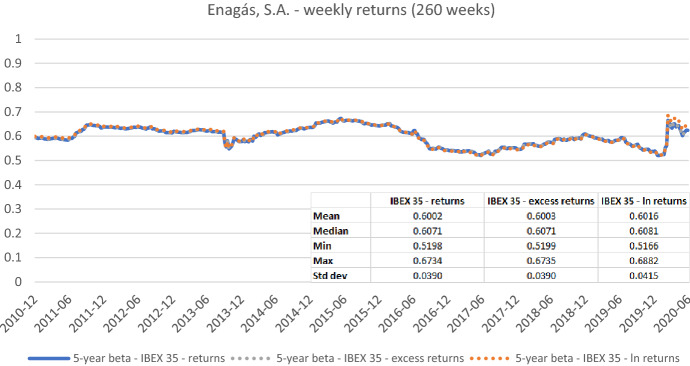
Betas for Enagás, S.A. based on (simple) returns, excess returns, and logarithmic returns. This chart shows five-year betas for Enagás, S.A. from the end of December 2010 to the end of June 2020. Betas are calculated using either weekly simple returns, weekly excess returns (i.e., simple returns in excess of the short-term Spanish treasury bill return), or weekly logarithmic returns. The IBEX 35 is used as the benchmark stock market index. The following descriptive statistics for each beta time series are reported accordingly: mean, median, minimum, maximum, and standard deviation

While some academic studies rely on beta estimates based on excess returns, practitioners usually prefer the use of returns without the risk-free rate adjustment. The difference between estimates based on the two approaches is typically negligible as the daily or weekly returns on short-term treasury bills are relatively small. In Fig. 1, we show the five-year weekly rolling betas for *Enagás* from the end of December 2010 to the end of June 2020 calculated using weekly returns and weekly excess returns. In this figure, we rely on the IBEX 35 as the local benchmark stock market index and on short-term (1–3 month) Spanish treasury bill yields which were converted to weekly returns and subtracted from the market and stock returns to obtain excess returns. As indicated by the graph and the corresponding descriptive statistics, such as the average betas for the full sample period (i.e., 0.6002 and 0.6003), we do not find any significant differences between estimates based on one or another approach.^9^ A similar conclusion applies to betas estimated using simple and logarithmic returns which are calculated with an adjusted price $P$ as follows:

3 $$\begin{gathered} {\text{Simple}}\quad R_{i,t} = { }\frac{{P_{i,t} }}{{P_{i,t - 1} }} - 1 \hfill \\ {\text{Logarithmic}}\quad R_{i,t} = ln\left( {P_{i,t} } \right) - ln\left( {P_{i,t - 1} } \right) \hfill \\ \end{gathered}$$

The differences between empirical estimations based on different return types are still debated in the academic literature (e.g., Hudson and Gregoriou 2015). In this context, logarithmic returns are typically assumed to suit the normal distribution assumption better and to mitigate the impact of extreme values on the results. As exemplarily shown in Fig. 1, the beta estimates only marginally differ (the average betas for *Enagás* using simple and logarithmic returns are 0.6002 and 0.6016, respectively).^10^

### Choice of index

**Fig. 2:**
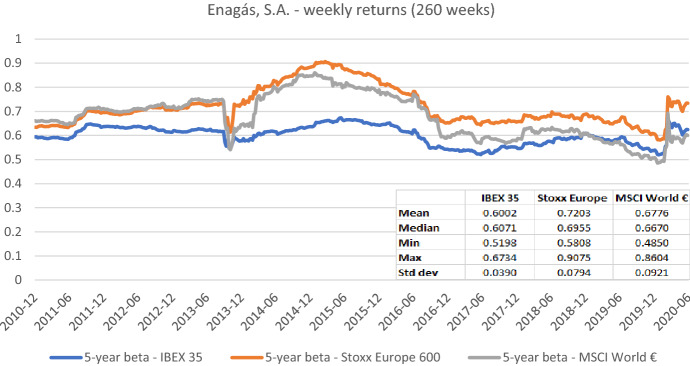
Betas for Enagás, S.A. based on the national, an international, and a global index. This chart shows five-year weekly betas for Enagás, S.A. from the end of December 2010 to the end of June 2020. Betas are calculated using either the IBEX 35, the Stoxx Europe 600, or the MSCI World € as the benchmark stock market index. The following descriptive statistics for each beta time series are reported accordingly: mean, median, minimum, maximum, and standard deviation

**Fig. 3:**
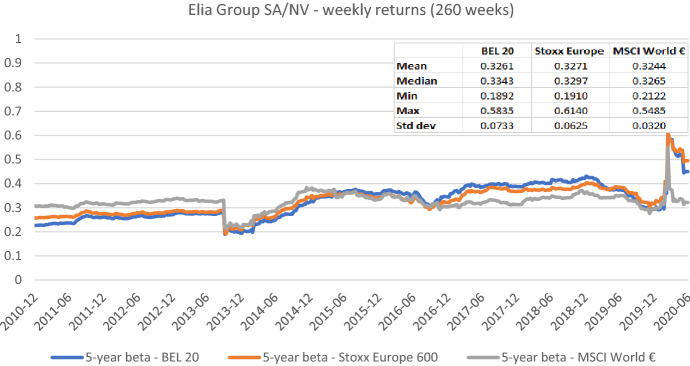
Betas for Elia Group SA/NV based on the national, an international, and a global index. This chart shows five-year weekly betas for Elia Group SA/NV from the end of December 2010 to the end of June 2020. Betas are calculated using either the BEL 20, the Stoxx Europe 600, or the MSCI World € as the benchmark stock market index. The following descriptive statistics for each beta time series are reported accordingly: mean, median, minimum, maximum, and standard deviation

**Fig. 4:**
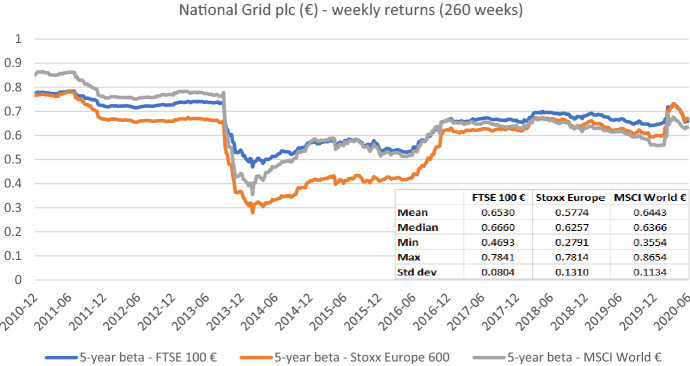
Betas for National Grid plc based on the national, an international, and a global index. This chart shows five-year weekly betas for National Grid plc from the end of December 2010 to the end of June 2020. Betas are calculated using either the FTSE 100 €, the Stoxx Europe 600, or the MSCI World € as the benchmark stock market index. The following descriptive statistics for each beta time series are reported accordingly: mean, median, minimum, maximum, and standard deviation

**Fig. 5:**
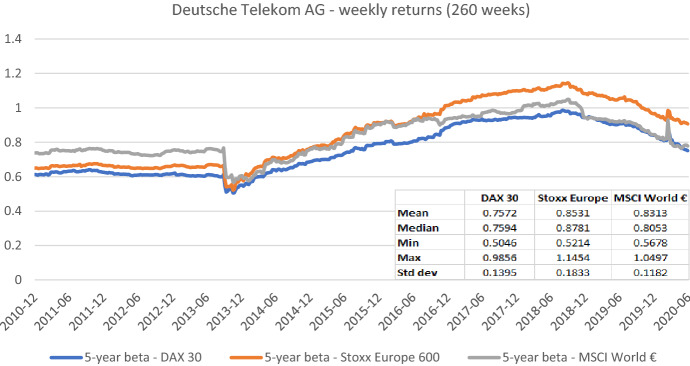
Betas for Deutsche Telekom AG based on the national, an international, and a global index. This chart shows five-year weekly betas for Deutsche Telekom AG from the end of December 2010 to the end of June 2020. Betas are calculated using either the DAX 30, the Stoxx Europe 600, or the MSCI World € as the benchmark stock market index. The following descriptive statistics for each beta time series are reported accordingly: mean, median, minimum, maximum, and standard deviation

**Fig. 6:**
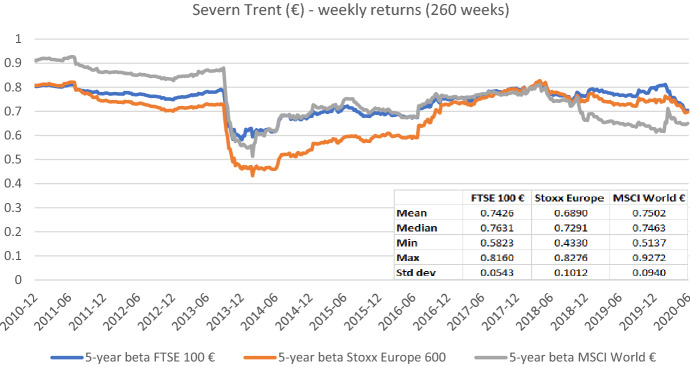
Betas for Severn Trent plc based on the national, an international, and a global index. This chart shows five-year weekly betas for Severn Trent plc from the end of December 2010 to the end of June 2020. Betas are calculated using either the FTSE 100 €, the Stoxx Europe 600, or the MSCI World € as the benchmark stock market index. The following descriptive statistics for each beta time series are reported accordingly: mean, median, minimum, maximum, and standard deviation

An important aspect of the beta estimation process is choosing the relevant market index to be used as a proxy for the market portfolio. With regard to the CAPM, Sharpe (1964) and Lintner (1965) do not define whether a national, a broader international (e.g., European), or even a worldwide market portfolio should be used. Grauer et al. (1976) spell out the assumptions that underly an international interpretation of the CAPM. Without going into detail, the choice of index depends on a clear definition of the market in which the CAPM is assumed to hold. In the context of regulatory studies, Stehle (2010) emphasizes that when betas are estimated for a sample of international firms, from the theoretical point of view, the capital markets should be assumed to be internationally or globally integrated, because the sample firm betas are only comparable and useful for further calculations in this case. Since all the firms in our study are from Europe, a broad European index denominated in euros appears to be most suitable (although a global index could also be used).^11^ For studies based only on firms from the same country, the choice of a national index may be a good approximation.^12^ Global studies which investigate firms from different international markets might also use the more sophisticated multifactor international CAPM of Solnik (1974, 1983) and Sercu (1980) that includes the exchange rate risk premia. However, the latter is more challenging to implement and thus might be less suited in the regulatory context. The impact of the market index choice on beta estimates is shown in Figs. 2, 3, 4, 5, and 6. In these figures, we plot the five-year betas of *Enagás, Elia Group, National Grid, Deutsche Telekom*, and *Severn Trent* which represent the different groups of regulated firms in our study.^13^ We use the major national indices, the Stoxx Europe 600 index, and the MSCI World index to proxy for national, international, and global market portfolios, respectively (see Table 1).

In sum, the graphs indicate that betas estimated with different market indices typically have similar patterns over the full sample period, but their level can differ noticeably at specific points in time. These results suggest that choosing the relevant market portfolio cannot be done randomly as it can significantly affect beta estimates. The decision to use a specific index must be theoretically underpinned.^14^

### Length of the estimation window

**Fig. 7:**
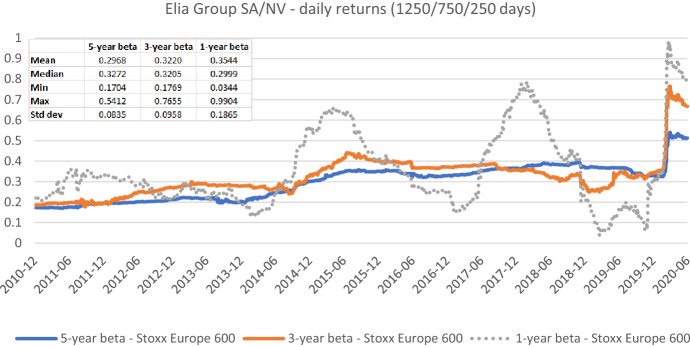
Betas for Elia Group SA/NV based on daily returns for five-, three-, and one-year estimation windows. This chart shows daily betas for Elia Group SA/NV from the end of December 2010 to the end of June 2020. Betas are calculated using either five-, three-, or one-year estimation window. The Stoxx Europe 600 is used as the benchmark stock market index. The following descriptive statistics for each beta time series are reported accordingly: mean, median, minimum, maximum, and standard deviation

**Fig. 8:**
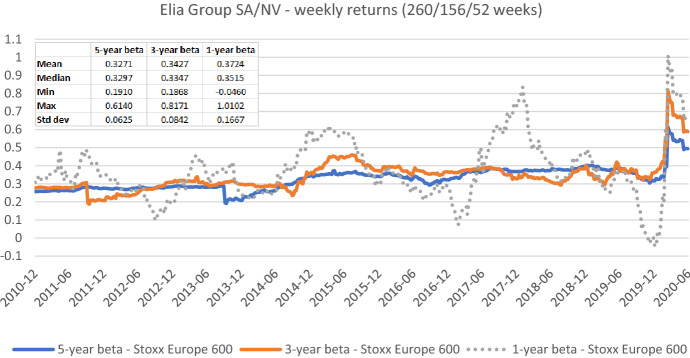
Betas for Elia Group SA/NV based on weekly returns for five-, three-, and one-year estimation windows. This chart shows weekly betas for Elia Group SA/NV from the end of December 2010 to the end of June 2020. Betas are calculated using either five-, three-, or one-year estimation window. The Stoxx Europe 600 is used as the benchmark stock market index. The following descriptive statistics for each beta time series are reported accordingly: mean, median, minimum, maximum, and standard deviation

**Fig. 9:**
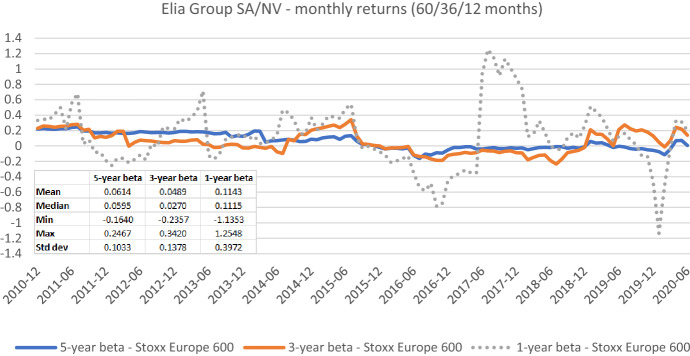
Betas for Elia Group SA/NV based on monthly returns for five-, three-, and one-year estimation windows. This chart shows monthly betas for Elia Group SA/NV from the end of December 2010 to the end of June 2020. Betas are calculated using either five-, three-, or one-year estimation window. The Stoxx Europe 600 is used as the benchmark stock market index. The following descriptive statistics for each beta time series are reported accordingly: mean, median, minimum, maximum, and standard deviation

**Fig. 10:**
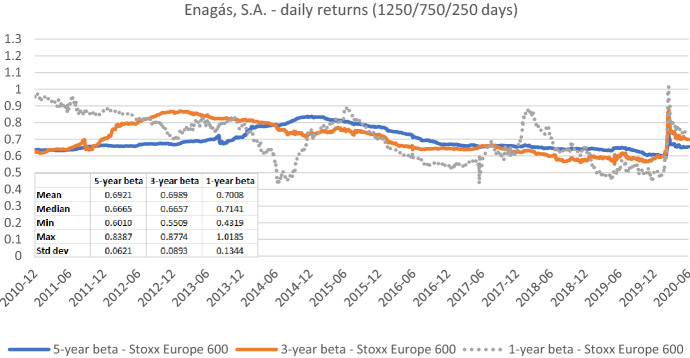
Betas for Enagás, S.A. based on daily returns for five-, three-, and one-year estimation windows. This chart shows daily betas for Enagás, S.A. from the end of December 2010 to the end of June 2020. Betas are calculated using either five-, three-, or one-year estimation window. The Stoxx Europe 600 is used as the benchmark stock market index. The following descriptive statistics for each beta time series are reported accordingly: mean, median, minimum, maximum, and standard deviation

**Fig. 11:**
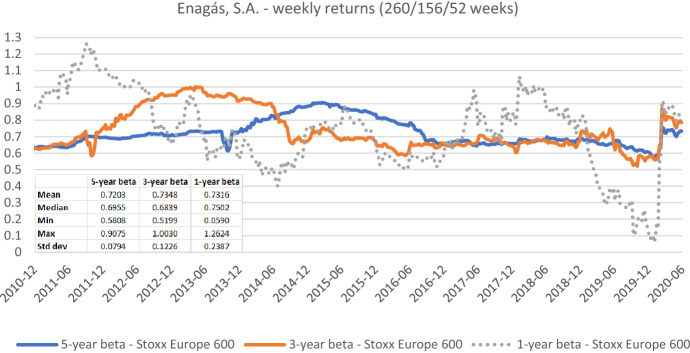
Betas for Enagás, S.A. based on weekly returns for five-, three-, and one-year estimation windows. This chart shows weekly betas for Enagás, S.A. from the end of December 2010 to the end of June 2020. Betas are calculated using either five-, three-, or one-year estimation window. The Stoxx Europe 600 is used as the benchmark stock market index. The following descriptive statistics for each beta time series are reported accordingly: mean, median, minimum, maximum, and standard deviation

**Fig. 12:**
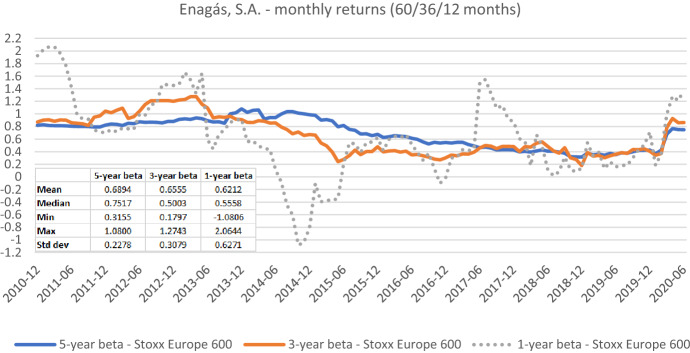
Betas for Enagás, S.A. based on monthly returns for five-, three-, and one-year estimation windows. This chart shows monthly betas for Enagás, S.A. from the end of December 2010 to the end of June 2020. Betas are calculated using either five-, three-, or one-year estimation window. The Stoxx Europe 600 is used as the benchmark stock market index. The following descriptive statistics for each beta time series are reported accordingly: mean, median, minimum, maximum, and standard deviation

The appropriate length of the estimation window—besides the suitable data frequency—is the most frequently discussed estimation parameter both in the literature and practice. In Figs. 7, 8, 9, 10, 11, 12, we exemplarily show the role of this issue for beta estimates by plotting the rolling betas of *Elia Group* and *Enagás* and reporting the relevant descriptive statistics. We report three graphs for each company to show, whether the window length (five-, three-, and one-year) affects betas in the same or in different ways for the alternative return intervals (daily, weekly, and monthly returns).

Here we rely only on the Stoxx Europe 600 index as our preferred market portfolio proxy. To plot betas calculated on a daily (weekly or monthly) basis over the full sample period (December 2010 to June 2020), we use the simplifying assumption that a year has 250 trading days (52 weeks and 12 months, respectively).

Estimation windows used to calculate CAPM betas range from a couple of months to several years, although the five-year period is most common in empirical studies (Groenewald and Fraser 2000). In the regulatory context, betas based on different window lengths are often combined in a judgmental process, but it is generally recognized that longer-term estimation windows of three or five years provide more stable beta forecasts.

Figures 7, 8, 9, 10, 11, 12 strongly support the latter: beta volatility (i.e., the standard deviation) for both sample firms *Elia Group* and *Enagás* increases with shorter estimation windows, irrespective of the data frequency used in the calculation. For instance, using daily returns, the standard deviation of *Elia Group* betas increases slightly from 0.0835 with the five-year estimation window to 0.0958 with the three-year estimation period. It doubles to 0.1865 with a one-year period. When we use weekly returns the volatility increase is similar as with daily data. Using monthly returns, the volatility increase is even larger as we go from five-year betas to one-year betas. Note however, that the monthly betas of *Elia Group* are much lower than the weekly or daily betas for all estimation windows. The latter are nearly identical for all windows.

The volatility results for *Enagás* are similar. Here the magnitude of the monthly betas is more in line with results based on daily or weekly betas.

Taken together our six graphs suggest that the five-year estimation window yields the most stable estimates over the full sample period, closely followed by the three-year estimation window. The application of the one-year estimation window is associated with a significantly higher beta volatility, which is at least nearly twice as high in all cases.

The graphs also show that the predictive power of one-year betas is rather weak. For example, *Elia’s* high weekly beta at year-end 2014 would have been a good prediction for a regulatory period lasting from June 2017 to June 2018, but not for earlier or later regulatory periods. As a consequence, using an average of one-, three-, and five-year betas, which is a widespread practice, also doesn’t seem to be a good idea.

As one-year betas seem to strongly and randomly fluctuate around their long-term mean over time, they are not well suited for the desirable beta stability in the regulatory context. Although our evidence suggests using a five-year estimation window for beta calculation, a shorter estimation period may be more appropriate when the required historical price data is missing or when the data from the more distant past is deemed misrepresentative for current (or future) risk estimates.

The graphs also show that it is prudent to look at different return intervals and estimation windows, even if concrete choices have been made in this regard.

### Return frequency

In the past, practitioners and scholars often used monthly returns for beta calculation, particularly because of data availability. More recent studies often suggest relying on daily returns (e.g., Levi and Welch 2017), which allow to estimate beta using more observations and, thus, can be associated with more accurate estimates. However, applying daily data assumes a sufficient liquidity of the respective securities. If this is not the case, such as for many smaller firms, arguments for weekly or monthly data can be made (Berk and DeMarzo 2020). Furthermore, lower-frequency returns are superior to daily data when the stock’s trading hours do not match those of the market index, which is often the case when international peer groups are used. Hence, there is a trade-off between possible beta underestimation with daily data due to infrequent or non-synchronous trading and less accurate estimates with lower-frequency data due to the smaller number of observations. In this context, estimates based on weekly returns provide a good compromise.

**Fig. 13:**
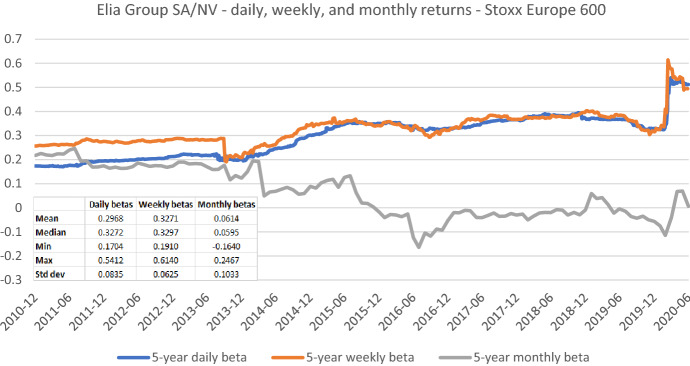
Betas for Elia Group SA/NV based on daily, weekly, and monthly returns. This chart shows five-year betas for Elia Group SA/NV from the end of December 2010 to the end of June 2020. Betas are calculated using either daily, weekly, or monthly returns. The Stoxx Europe 600 is used as the benchmark stock market index. The following descriptive statistics for each beta time series are reported accordingly: mean, median, minimum, maximum, and standard deviation

**Fig. 14:**
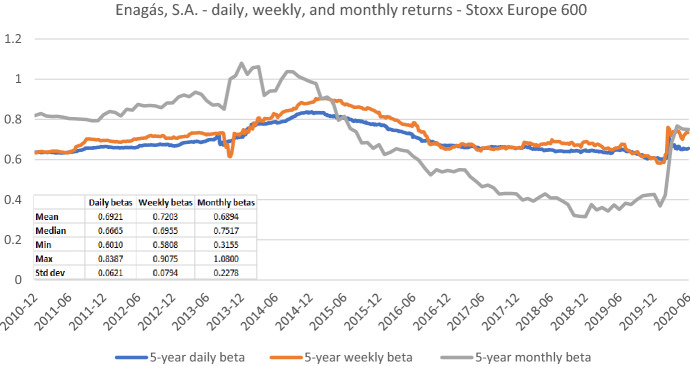
Betas for Enagás, S.A. based on daily, weekly, and monthly returns. This chart shows five-year betas for Enagás, S.A from the end of December 2010 to the end of June 2020. Betas are calculated using either daily, weekly, or monthly returns. The Stoxx Europe 600 is used as the benchmark stock market index. The following descriptive statistics for each beta time series are reported accordingly: mean, median, minimum, maximum, and standard deviation

In Figs. 13 and 14, we look at the same beta time series for *Elia Group* and *Enagás* as in the previous section, but from a different perspective. Because we focus on five-year betas, the range of the y-axis is much smaller and thus more precise.

Around 2010, *Elia Group*, was often assumed to have a low liquid stock.^15^ Frontier Economics (2012) explained its low beta as being a result of the Belgian regulatory system, which, in their judgement, guarantees bond-like returns. As a consequence, Frontier Economics did not include it in their sample. In 2010, *Elia Group* took over 60% of the large German transmission system operator *50Hertz*. Since then it certainly deserves to be considered a normal European electricity network operator and has been included in all regulatory cost of capital studies, including those by Frontier Economics after 2012.

Figure 13 shows that the beta estimates for *Elia Group* based on daily and weekly returns are nearly identical after 2013. Before 2013, the daily beta is considerably lower, which we attribute to the low liquidity. As mentioned above, the betas for *Elia Group* based on monthly returns substantially deviate from betas calculated with daily and weekly data. They are much lower and have a higher volatility. In line with that, Wright et al. (2018) report that betas based on monthly data may be subject to significant impacts of single returns due to the small number of observations. Thus, monthly betas can noticeably jump from month to month. Wright et al. (2018, G-139) also note "*that betas appear to fall at lower frequencies*" and, thus, noticeably undercut their higher-frequency counterparts.^16^

Our findings for *Enagás* (in Fig. 14) support these observations, in particular, for monthly betas calculated for the last five years.^17^In this regard, the recent study by Agrrawal et al. (2022) also shows that betas estimated using higher frequency returns (i.e., daily and weekly returns) have a better predictive power—based on tracking errors—compared to those calculated using monthly returns.

An additional issue with monthly data refers to different time points that can be used for the return calculation. We calculate monthly returns based on last trading days in the respective months, although the employment of mid-month returns is also conceivable. Stehle (2010) shows that the two alternatives can result in substantially different beta estimates. This issue is also theoretically applicable to weekly data as returns could be calculated based on each of the five working days. However, Friday returns appear to be more common.^18^

To summarize, our results suggest that weekly betas seem to be best suited for smaller firms and international peer groups to avoid issues related to infrequent or non-synchronous trading. In contrast, daily betas appear to be more suitable for larger firms that are listed on the same stock exchange. Monthly betas differ significantly from their daily and weekly counterparts and are subject to larger fluctuations, possibly due to the small number of observations used for estimation. Based on these arguments, we recommend weekly betas in pan-European regulatory contexts.

Dimson (1979) suggests an alternative approach for dealing with stock illiquidity and non-synchronous trading: applying leads (*t* + *1*) and lags (*t − 1*) of market returns in the scope of beta estimation. This is due to the fact that new market information is typically incorporated faster in the prices of liquid stocks and slower in the prices of illiquid ones, which can cause “simple” betas to be systematically over- or underestimated.^19^ The Dimson-beta is calculated as the sum of the three beta coefficients:

4 $$R_{i,t} = \alpha_{i} + \beta_{i}^{a} R_{m,t - 1} + \beta_{i}^{b} R_{m,t} + \beta_{i}^{c} R_{m,t + 1} + {\upvarepsilon }_{{{\text{i}},{\text{t}}}} \;und\;\beta_{i}^{{{\text{Dimson}}}} = \beta_{i}^{a} + \beta_{i}^{b} + \beta_{i}^{c}$$

However, in our context, the application of the Dimson approach appears to be unnecessary as the weekly data frequency already mitigates the issues mentioned above. Moreover, the application of such a lead-lag structure can add further noise to beta estimates (Hollstein 2020). To our knowledge, this approach has not been used in the regulatory context to date.

### Sudden increases and decreases

The standard corporate finance textbooks, such as Berk and DeMarzo (2020) or Ross et al. (2019), emphasize that betas can vary over time not only due to corporate changes, but also because of unusual market conditions as in the case of the tech bubble (1998 to 2001) or the financial crisis (2008 to 2009). According to Berk and DeMarzo (2020), the extreme stock and market movements during such turbulent times could, on the one hand, be treated as outliers. On the other hand, they may constitute a valuable opportunity to assess the stock’s sensitivity to future shocks. Several studies have shown that the financial crisis had a significant impact on beta estimates, which can be attributed to a substantial increase of stock market volatility that, in turn, led to an increase of the systematic risk for a majority of companies (e.g., Ben Slimane et al. 2017; Bellelah et al. 2017). In other words, the betas of very large companies decreased, while the betas of mid-size and small companies increased, on average.^20^ Grout and Zalewska (2016) further argue that changes in the systematic risk of different market sectors could be attributed to spill-over effects from the banking industry that was particularly affected by the crisis.

**Fig. 15:**
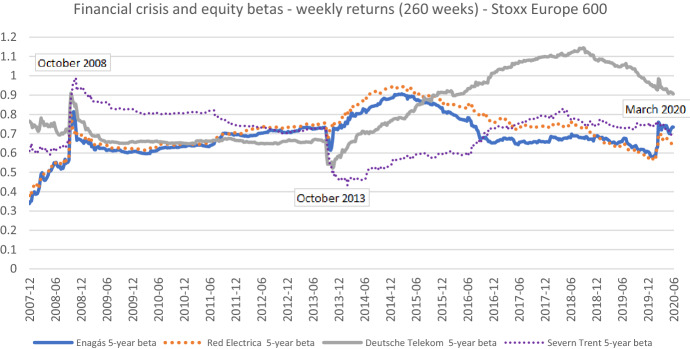
Financial crisis and betas for different regulated firms based on weekly returns—extended sample length. This chart shows five-year weekly betas for Enagás, S.A, Red Electrica Corporacion S.A., Deutsche Telekom AG, and Severn Trent plc from the end of December 2007 to the end of June 2020. The Stoxx Europe 600 is used as the benchmark stock market index. October 2008 is the month when the stock market crash associated with the global financial crisis occurred. October 2013 refers to the timepoint when the estimation window used for the beta calculation does no longer include the extreme returns associated with the stock market crash. March 2020 is the month when the largest stock market drop associated with the COVID-19 pandemic occurred

As regulated firms are frequently assumed to be less sensitive to economic shocks, we first assess whether the patterns ascertained in the literature also occur in our sample. Therefore, we extend the sample length back to 2007 for four sample firms which together represent all of our sectors of interest and for which the required return data is available. The results are found in Fig. 15. As the graph shows, the betas of all four regulated firms increased considerably in October 2008 when the global market crash occurred.^21^ Subsequently, in the course of the next months or years, betas reverted to the pre-crash levels, at least to some extent. Thus, crisis-related beta spikes also show up for regulated firms.

**Fig. 16:**
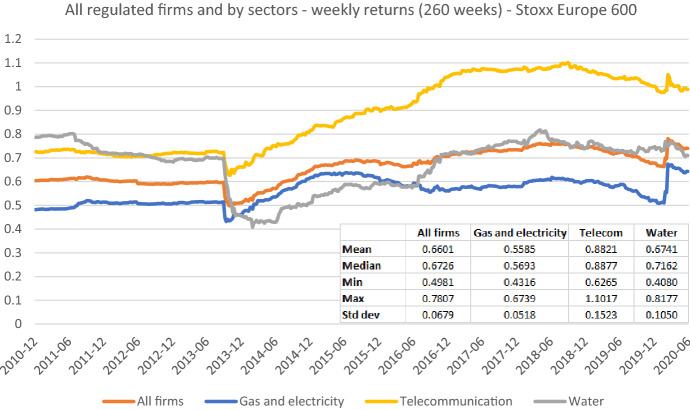
Average betas for all regulated firms and by sectors based on weekly returns. This chart shows average five-year weekly betas for all sample firms, seven gas and electricity network operators, three telecommunication network operators, and two water utilities from the end of December 2010 to the end of June 2020, respectively. The Stoxx Europe 600 is used as the benchmark stock market index. The following descriptive statistics are reported accordingly: mean, median, minimum, maximum, and standard deviation

More importantly, Fig. 15 shows a noticeable beta dip in October 2013, exactly five years after the increase in 2008. The sharp beta decrease and its subsequent reversal are related to the crisis in 2008, but they are mainly of a technical nature. Betas substantially dropped in October 2013 because the extreme returns of the 2008 stock market crash are no longer included in the observation period of the beta estimation. We find a similar pattern, when we average over all twelve sample firms as shown in Fig. 16. This finding is further substantiated by the corresponding figures for each sample firm, which we provide, in addition to those already reported, in appendix 18, 19, 20, 21, 22, 23, 24.

To assess whether the technical beta decrease is also significant in a statistical sense, we also compare the average betas of our sample firms at the end of October 2013 (i.e., without extreme returns in the estimation window) with those at the end of September 2013 (i.e., with extreme returns). The results on the mean differences between these two months are reported in Table 2. The paired *t*-test indicates that the negative difference of −0.0987 between the average betas in October (0.4986) and in September (0.5972) is highly significant (*t* = −6.202) and this result is further corroborated by the Wilcoxon signed-rank test (*z* = −3.059).

**Table 2:** Betas after the global financial crisis—the technical effect in 2013

Weekly returns (260 weeks)	End of October 2013	End of September 2013
Market portfolio: Stoxx Europe 600	(10/25/2013)	(9/27/2013)
Elia Group SA/NV	0.2139	0.2879
Red Electrica Corporacion S.A	0.7191	0.7548
Terna—Rete Elettrica Nazionale S.p.A	0.3642	0.4243
Enagás, S.A	0.6170	0.7297
Snam S.p.A	0.3005	0.3192
Redes Energeticas Nacionais SGPS S.A	0.3504	0.4252
National Grid plc	0.4802	0.6597
Deutsche Telekom AG	0.5435	0.6664
Vodafone Group plc	0.5819	0.6939
Telefónica S.A	0.7577	0.8097
Severn Trent plc	0.5461	0.7293
United Utilities Group plc	0.5084	0.6667
Mean	0.4986	0.5972
Difference	− 0.0987	
Paired *t* test	*t* = − 6.202***	
Wilcoxon signed-rank test	*z* = − 3.059***	

This table reports betas of our sample firms at the end of September 2013 and at the end of October 2013. The mean differences between them are reported accordingly. The estimation window used to calculate betas at the end of September 2013 covers extreme returns associated with the stock market crash in October 2008, while the estimation window of betas at the end of October 2013 does no longer include them. For both dates, betas are calculated using a five-year estimation window, weekly returns, and the Stoxx Europe 600 as the benchmark stock market index. The paired *t*-test and the Wilcoxon signed-rank test are used to assess statistical significance of the mean differences. ***, **, and * denote statistical significance at the 1%-, 5%-, and 10%-level, respectively

Similar to the patterns ascertained during the financial crisis, betas tend to revert after a sharp drop. Such reversals seem to be in line with the findings of Patton and Verardo (2012) who show that after an increase related to firm-specific news betas tend to rapidly revert to their long-term averages. Furthermore, our findings significantly extend the empirical evidence on the effect of the Brexit referendum on betas of British firms provided by NERA (2018). The latter study shows that betas of certain British telecommunication operators increased immediately after the Brexit referendum in June 2016 and decreased after the respective observations fell out of the estimation window. Betas of British regulated utilities were unaffected by the referendum. In contrast, we show that the global financial crisis has noticeably affected betas of all regulated firms alike and the sudden increases and decreases typically reverse within short time periods, at least to some extent.

Because we use a five-year estimation window in our main analyses, the beta dips occur five years after the global market crash. Obviously, using an alternative estimation window, the dips would emerge at a different point in time. In the case of a three-year estimation window, it would be October 2011. Furthermore, we also see dips with daily data, but they are not as sharp because the extreme returns are distributed over multiple days rather than single weeks (see, for instance, Fig. 10).

In the regulatory context, beta spikes and dips can be an issue when the estimation cut-off date lies on or near the top of the beta spikes (or bottom of the dips) and the regulatory period starts one or two years later. The results suggest that it is indispensable to look at beta times series for several years to identify beta trends over a longer time period.

Our findings with respect to the effects of financial crisis on beta estimates have important implications for future regulatory and academic studies because we find similar patterns also in the recent coronavirus crisis which may have an impact on betas at least in the next couple of years. As can be seen in almost all figures in this paper, the COVID-19-related shock resulted in a substantial (at least temporarily) increase in beta estimates that can be particularly attributed to the stock market crash in February and March 2020.^22^ To illustrate how this particular shock affected beta estimates in the short run, we compare betas in the week of the largest stock market drop (i.e., at the end of the calendar week 11) with beta estimates before the beginning of the crash (i.e., at the end of the calendar week 8). The results are reported in Table 3. In Table 4, we assess the sensitivity of stock returns to market returns in the week of the major stock market crash to explain the differences in beta estimates across firms. Finally, to evaluate how the coronavirus crisis affected beta estimates in the long run, we look at the differences between recent betas after a market upswing and betas from the last three years. The results are reported in Table 5.

**Table 3:** Betas and the coronavirus crisis—the short-term view

Weekly returns (260 weeks)	End of week 11 in 2020	End of week 8 in 2020
Market portfolio: Stoxx Europe 600	(3/13/2020)	(2/21/2020)
Elia Group SA/NV	0.6140	0.3362
Red Electrica Corporacion S.A	0.6919	0.5672
Terna—Rete Elettrica Nazionale S.p.A	0.6580	0.5247
Enagás, S.A	0.7601	0.5859
Snam S.p.A	0.7473	0.5628
Redes Energeticas Nacionais SGPS S.A	0.5423	0.3880
National Grid plc	0.7038	0.6019
Deutsche Telekom AG	0.9844	0.9397
Vodafone Group plc	1.0342	0.8885
Telefónica S.A	1.1338	1.0967
Severn Trent plc	0.7530	0.7418
United Utilities Group plc	0.7451	0.7225
Mean	0.7807	0.6630
Difference	0.1177	
Paired *t* test	*t* = 5.178***	
Wilcoxon signed-rank test	*z* = 3.059***	

This table reports betas of our sample firms at the end of the calendar week 8 in 2020 and at the end of the calendar week 11 in 2020. The mean differences between them are reported accordingly. The calendar week 8 in 2020 refers to the time before the start of the COVID-19-related stock market crash. The calendar week 11 in 2020 is the week when the largest stock market drop associated with the COVID-19 pandemic occurred. For both dates, betas are calculated using a five-year estimation window, weekly returns, and the Stoxx Europe 600 as the benchmark stock market index. The paired *t*-test and the Wilcoxon signed-rank test are used to assess statistical significance of the mean differences. ***, **, and * denote statistical significance at the 1%-, 5%-, and 10%-level, respectively

**Table 4:** Individual stock returns during the coronavirus-related market crash

Weekly returns	Week 11 in 2020
Market portfolio: Stoxx Europe 600	3/6/2020 to 3/13/2020
	Stock return divided by market return
Elia Group SA/NV	1.2995
Red Electrica Corporacion S.A	0.9328
Terna—Rete Elettrica Nazionale S.p.A	0.9523
Enagás, S.A	1.1894
Snam S.p.A	1.2038
Redes Energeticas Nacionais SGPS S.A	0.8786
National Grid plc	0.9556
Deutsche Telekom AG	1.1546
Vodafone Group plc	1.3700
Telefónica S.A	1.2892
Severn Trent plc	0.7553
United Utilities Group plc	0.6922

This table reports stock return to market return ratios for our sample firms at the end of the calendar week 11 in 2020. The calendar week 11 in 2020 is the week when the largest stock market drop associated with the COVID-19 pandemic occurred. Stock return to market return ratios are calculated by dividing weekly stock returns by the weekly stock return of the Stoxx Europe 600 index. Weekly returns are calculated from Friday, 6th March 2020 to Friday, 13th March 2020

**Table 5:** Betas and the coronavirus crisis—the long-term view

Weekly returns (260 weeks)	End of June 2020	End of June 2019	End of June 2018	End of June 2017	Average
Market portfolio: Stoxx Europe 600	(06/26/2020)	(06/28/2019)	(06/29/2018)	(06/30/2017)	2017–2019
Elia Group SA/NV	0.4947	0.3843	0.3739	0.3659	0.3747
Red Electrica Corporacion S.A	0.6478	0.6500	0.7371	0.7349	0.7073
Terna S.p.A	0.6600	0.6030	0.6154	0.5875	0.6020
Enagás, S.A	0.7337	0.6588	0.6791	0.6566	0.6649
Snam S.p.A	0.7414	0.6457	0.6660	0.6011	0.6376
Redes Energeticas S.A	0.5516	0.5304	0.5084	0.4601	0.4996
National Grid plc	0.6698	0.6217	0.6678	0.6279	0.6391
Mean	0.6427	0.5848	0.6068	0.5763	0.5893
	2020—2019				2020—Avr
Difference	0.0579				0.0534
Paired *t* test	*t* = 3.842***				*t* = 2.417*
Wilcoxon signed-rank test	*z* = 2.197**				*z* = 1.690*
Deutsche Telekom AG	0.9061	1.0546	1.1178	1.0754	1.0826
Vodafone Group plc	0.9601	0.9331	0.9461	0.9374	0.9389
Telefónica S.A	1.0985	1.1237	1.1697	1.2141	1.1692
Mean	0.9882	1.0372	1.0779	1.0756	1.0636
	2020—2019				2020—Avr
Difference	−0.0489				−0.0753
Paired *t* test	*t* = −0.940				*t* = −1.319
Wilcoxon signed-rank test	*z* = −0.535				*z* = −1.069
Severn Trent plc	0.6979	0.7273	0.7748	0.7564	0.7528
United Utilities Group plc	0.7217	0.7308	0.7716	0.7223	0.7415
Mean	0.7098	0.7290	0.7732	0.7393	0.7472
	2020—2019				2020—Avr
Difference	−0.0192				−0.0373
Paired *t* test	*t* = −1.896				*t* = −2.128
Wilcoxon signed-rank test	*z* = −1.342				*z* = −1.342

This table reports betas of our sample firms from the end of June 2017 to the end of June 2020. The mean differences between betas at the end of June 2020 and at the end of June 2019 (Column 2), and between betas at the end of June 2020 and average betas for the period 2017 to 2019 (Column 6) are reported accordingly by industry sectors. All betas are calculated using a five-year estimation window, weekly returns, and the Stoxx Europe 600 as the benchmark stock market index. The paired *t*-test and the Wilcoxon signed-rank test are used to assess statistical significance of the mean differences. ***, **, and * denote statistical significance at the 1-, 5-, and 10%-level, respectively

Table 3 shows that the average beta of all stocks in our sample increased significantly (by 0.1177) at the end of the week with the largest stock market drop (i.e., the calendar week 11 in 2020) compared to the average beta three weeks before. Furthermore, betas increased for all individual sample firms, regardless of the specific sector to which they belong, so that besides the *t*-test (*t* = 5.178) also the Wilcoxon signed-rank test (*z* = 3.059) indicates a high statistical significance in addition to the economic significance. The conclusion is that the economic shock related to the coronavirus pandemic had a significant impact on the betas of the stocks in our sample in the short run. However, it must be noted that the magnitude of this effect varies across firms. In particular, the betas of both water utilities—*Severn Trent* and *United Utilities Group*—increased only marginally. In this context, the results in Table 4 suggest that the latter finding can be attributed to the substantially lower stock return to market return ratios for both firms in the week of the major market crash. Thus, while the negative stock returns of other sample firms were either similar or more pronounced compared to the negative market return (i.e., stock return to market return ratio is either roughly equal to or above one), the negative stock returns of water utilities were noticeably smaller with ratios equal to 0.7553 and 0.6922, respectively. In addition, our results indicate that the beta increase is less pronounced for stocks that already have a beta near one compared to low-beta stocks. That is even the case if they have similar stock return to market return ratios, such as *Telefónica* and *Elia Group*.

The evidence of a longer-term effect of the coronavirus crisis provided in Table 5 is mixed. Considering only the group of electricity and gas network operators on the top of the table, we find that their average beta at the end of June 2020 (i.e., approximately three months after the stock market crash) was still significantly higher (by 0.0579) with respect to the average beta one year before.^23^ In addition, we compare the average beta of these firms in June 2020 with the long-term average of sample betas calculated using the last three years. As these betas were relatively stable in the past, we still find a significant difference of 0.0534 between estimates before and after the market crash in 2020.

On the other hand, looking at the groups of telecommunication operators and water utilities separately, we find that their average betas decreased in the long run since 2019. However, this result can be partly attributed to the slightly negative beta trends for *Deutsche Telekom* and *Severn Trent* in the last few years. Nevertheless, the corresponding graphs for telecommunication network operators (see Figs. 5, 22, and 23) and for water utilities (see Figs. 6 and 24) suggest that the coronavirus crisis had mainly a short-term effect on betas of these sectors. In line with the findings from the previous section, the aforementioned difference between electricity and gas network operators and, in particular, their telecom counterparts can be related to the fact that the latter already have higher average betas near one, so that short-term market fluctuations do not sustainably affect their systematic risk.

To summarize, the results indicate that the betas of all regulated firms examined in our study were at least temporarily affected by the coronavirus shock. Hence, researchers and scholars looking at regulated firms in the future should be aware of beta dips that can occur when their estimation windows no longer include the extreme returns related to the market crash in February and March 2020. To address this issue, an adjustment of the estimation window by shifting it for- or backward could be useful. Furthermore, our findings suggest that due to periods of high stock volatility occurring in the context of different economic crises, the homoscedasticity assumption of the OLS estimator can be violated. Thus, future regulatory studies may consider more sophisticated models for beta estimation, such as the Generalized Least Squares (GLS) technique, that account for a non-constant variance of residuals.

## Blume and Vasicek adjustments

To alleviate the impact of estimation errors on beta values as well as to address their temporal instabilities, prior studies suggest applying post-estimation adjustments. One of them was suggested by Blume (1971, 1975) who identifies a tendency of beta estimates to move over time toward the market average, which is equal to one. Thus, estimated betas over (under) the value of one have a higher probability to be subject to overestimation (underestimation) error. To reduce such an error, Blume suggests adjusting the beta forecast for the next time period (*t* + *1*) as follows:

5 $$\beta_{i,t + 1} = {\text{a}} + {\text{b*}}\beta_{i,t}$$

In practice, 1/3 and 2/3 are the common values used for $\mathrm{a}$ and $\mathrm{b}$ parameters, which were estimated by Blume using U.S. stock market data between 1926 and 1968.

The Blume adjustment is less popular in the regulatory context than the approach suggested by Vasicek (1973) which is frequently used to adjust betas towards one. The Vasicek approach, which is based on Bayesian inference, calculates a beta forecast for the next period as a weighted average of the historical beta of a relevant portfolio ${\beta }_{p}$ and the estimated stock beta ${\beta }_{i,t}^{OLS}$:

6 $$\beta_{i,t + 1} = \beta_{p} \left( {1 - x_{i} } \right) + x_{i} \beta_{i,t}^{{{\text{OLS}}}} \;{\text{with}}\; x_{i} = \frac{{{\text{VAR}} \left( {\beta_{p} } \right)}}{{{\text{VAR}} \left( {\beta_{p} } \right) + {\text{SE}}^{2} \left( {\beta_{i,t}^{{{\text{OLS}}}} } \right)}}$$

Vasicek (1973) argues that when the firms’ industry beta is lower or higher than the average market beta of one, the relevant industry should be used as the benchmark portfolio ${\beta }_{p}$. Lally (1998) also finds that adjusting betas towards the industry beta of the respective stock is superior to the simple adjustment toward one.^24^ To visualize this issue, in Fig. 16 we show the temporal development of average betas for all of our sample firms as well as for different groups of regulated firms separately. All equity betas are calculated using weekly trade-to-trade total returns and the Stoxx Europe 600 as the market portfolio. We use the five-year estimation window for all stocks.^25^

Figure 16 shows that the long-term average of beta estimates for all regulated firms in our sample equals 0.6601 and is, thus, far below the hypothetical market value of one. Furthermore, when allocating firms to industry sectors, it becomes apparent that closely related gas and electricity network operators have even much lower average beta (0.5585). In contrast, the respective average for the telecommunication network operators is substantially higher (0.8821) lying above or below the value of one during the last ten years. The average beta of water utilities (0.6741) is, in turn, nearly equal to the sample mean.

**Fig. 17:**
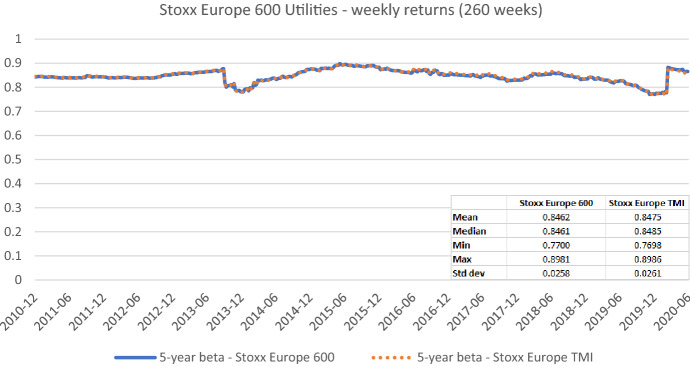
Betas for the Stoxx Europe 600 Utilities index based on the Stoxx Europe 600 index and the Stoxx Europe TMI. This chart shows average five-year weekly betas for the Stoxx Europe 600 Utilities index from the end of December 2010 to the end of June 2020. The Stoxx Europe 600 index and the Stoxx Europe TMI index are used as the benchmark, respectively. The following descriptive statistics for each beta time series are reported accordingly: mean, median, minimum, maximum, and standard deviation

**Fig. 22:**
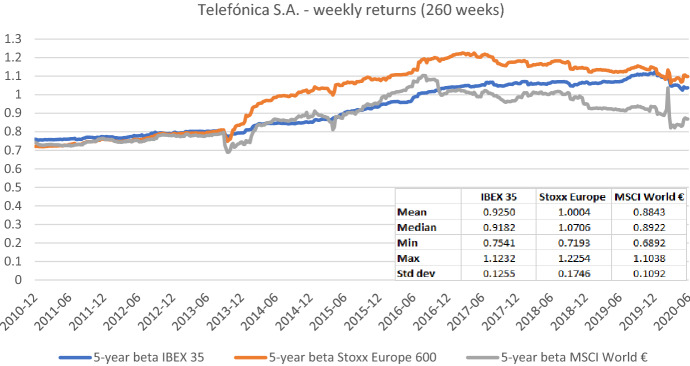
Betas for Telefónica S.A. based on the national, an international, and a global index. This chart shows five-year weekly betas for Telefónica S.A. from the end of December 2010 to the end of June 2020. Betas are calculated using either the IBEX 35, the Stoxx Europe 600, or the MSCI World € as the benchmark stock market index. The following descriptive statistics for each beta time series are reported accordingly: mean, median, minimum, maximum, and standard deviation

**Fig. 23:**
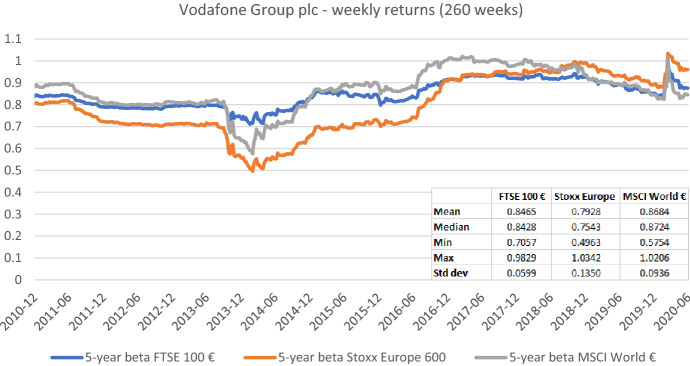
Betas for Vodafone Group plc based on the national, an international, and a global index. This chart shows five-year weekly betas for Vodafone Group plc from the end of December 2010 to the end of June 2020. Betas are calculated using either the FTSE 100 €, the Stoxx Europe 600, or the MSCI World € as the benchmark stock market index. The following descriptive statistics for each beta time series are reported accordingly: mean, median, minimum, maximum, and standard deviation

**Fig. 24:**
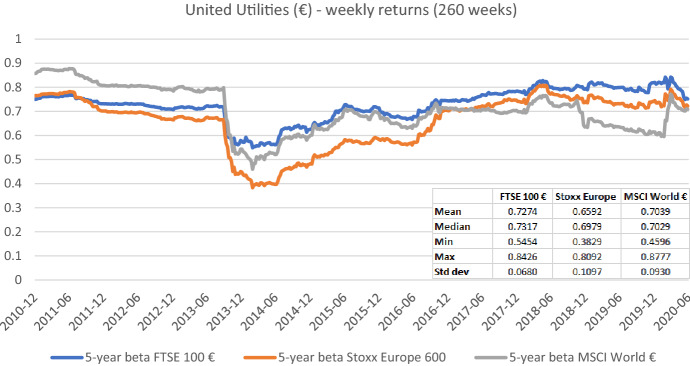
Betas for United Utilities plc based on the national, an international, and a global index. This chart shows five-year weekly betas for United Utilities plc from the end of December 2010 to the end of June 2020. Betas are calculated using either the FTSE 100 €, the Stoxx Europe 600, or the MSCI World € as the benchmark stock market index. The following descriptive statistics for each beta time series are reported accordingly: mean, median, minimum, maximum, and standard deviation

In Fig. 17, we further substantiate these results by looking at the betas of the Stoxx Europe 600 Utilities subindex that contains most of our European sample of nearly pure network operators plus many other regulated firms—except for the telecom operators. We rely on the Stoxx Europe 600 index which up to now we have always used as market portfolio and use the Stoxx Europe Total Market Index as an additional market portfolio for robustness purposes. Figure 17 shows that betas of utility firms are very stable over time, irrespective of the preferred benchmark portfolio. Their betas slightly fluctuate between values of 0.7700 (0.7698) and 0.8981 (0.8986) during the last ten years. Their long-term average equals 0.8462 (0.8475) and is therefore noticeably below one. Overall, due to the stability of regulated activities, a common practice of adjusting betas towards one appears to be inappropriate in our context. It could actually result in unduly high estimates rather than in reducing the estimation error. Furthermore, a beta increase over time can often be related to the fact that a regulated firm has added new non-regulated or international activities to its operations. Thus, a proper screening and selection of the relevant peer group firms shall be preferred to the mentioned post-estimation adjustments towards any predefined values.

## Conclusion

In this paper, we assess the major issues related to the process of beta estimation in academic studies and in practice using a sample of European network operators. We focus on a small number of individual stocks and small peer groups consisting of these stocks to closely examine their long beta time series. We look at all steps of beta calculation starting with data selection and cleaning procedures and finishing with post-estimation adjustments.

We first point out that total returns—returns adjusted for dividends and further corporate actions—should be used instead of raw stock and index returns. We also emphasize that all returns should be expressed in the same currency. And we suggest using trade-to-trade returns to avoid issues associated with non-trading days. Our results additionally show that it doesn’t actually matter for our estimates whether we use returns or excess returns or simple or logarithmic returns.

Furthermore, we stress that the relevant market portfolio used to calculate betas cannot be chosen randomly. Our evidence indicates that the use of different international and local indices often results in similar betas, but it can matter for certain firms and estimation periods. We suggest using an international index when investigating a sample of firms located in different countries.

In our study, five-year betas are the most stable, closely followed by three-year betas. We advise against the use of one-year betas in the regulatory context due to their strong and random fluctuation over time. Similar caution applies to monthly betas, which often significantly differ from daily and weekly betas. We recommend using weekly betas for international firms and smaller, less liquid stocks. We suggest the use of daily betas for larger companies listed on the same stock exchange.

The most important conclusion of our paper is that it is indispensable to look at beta times series and not only at the beta of a single date. Since significant sudden beta increases and decreases occur which often at least partially revert within short time periods, point estimates can be substantially distorted. We first show that substantial beta spikes occur during the 2008 worldwide economic crisis. More importantly, we find significant beta dips several years after this crisis when the related observations fall out of the estimation window. This finding has important implications for future studies as we also find a noticeable beta increase during the recent coronavirus crisis in 2020. Thus, researchers and practitioners should be aware of beta dips that can occur when the coronavirus-related observations are no longer included in the estimation window. We note that the aforementioned effects can differ across industries.

Finally, we show that in our context Blume and Vasicek adjustments towards one lead to overstated beta estimates. This is due to the fact that regulated activities are stable over time and the long-term average of beta estimates for regulated firms is noticeably below one.

In sum, our study provides important implications for practitioners and scholars and it stresses the necessity of a thorough analysis of beta trends in the past to avoid distorted estimates for the future.

## Figures and Tables

**Fig. 18:**
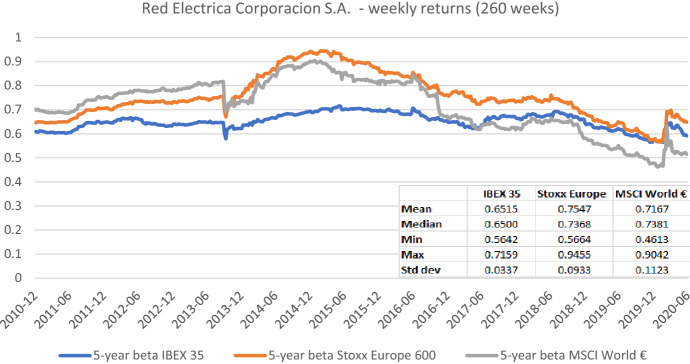
Betas for Red Electrica Corporacion S.A. based on the national, an international, and a global index. This chart shows five-year weekly betas for Red Electrica Corporacion S.A. from the end of December 2010 to the end of June 2020. Betas are calculated using either the IBEX 35, the Stoxx Europe 600, or the MSCI World € as the benchmark stock market index. The following descriptive statistics for each beta time series are reported accordingly: mean, median, minimum, maximum, and standard deviation

**Fig. 19:**
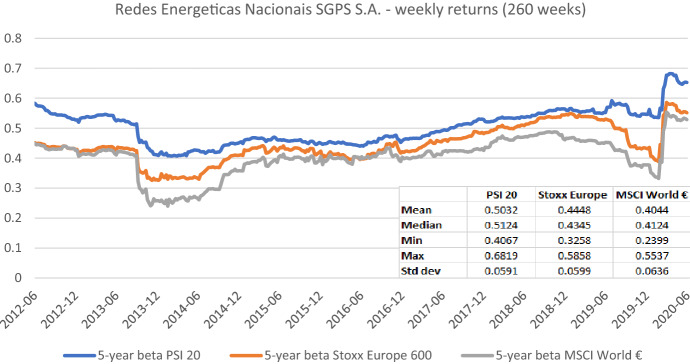
Betas for Redes Energeticas Nacionais SGPS S.A. based on the national, an international, and a global index. This chart shows five-year weekly betas for Redes Energeticas Nacionais SGPS S.A. from the end of December 2010 to the end of June 2020. Betas are calculated using either the PSI 20, the Stoxx Europe 600, or the MSCI World € as the benchmark stock market index

**Fig. 20:**
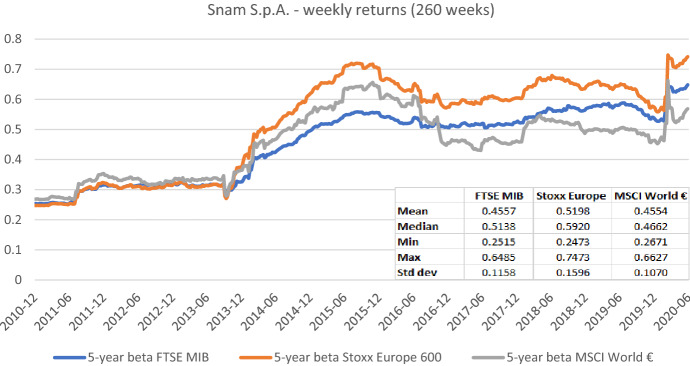
Betas for Snam S.p.A. based on the national, an international, and a global index. This chart shows five-year weekly betas for Snam S.p.A. from the end of December 2010 to the end of June 2020. Betas are calculated using either the FTSE MIB, the Stoxx Europe 600, or the MSCI World € as the benchmark stock market index. The following descriptive statistics for each beta time series are reported accordingly: mean, median, minimum, maximum, and standard deviation

**Fig. 21:**
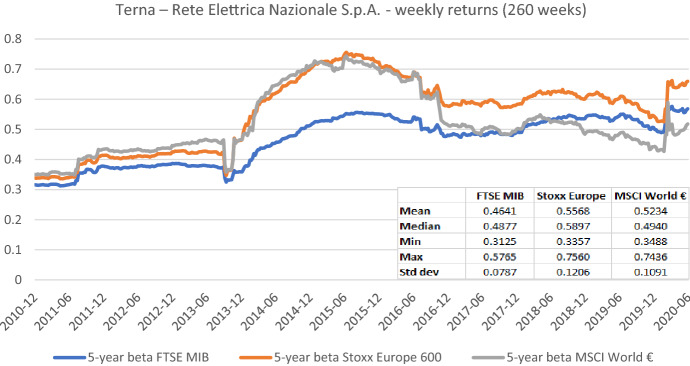
Betas for Terna—Rete Elettrica Nazionale S.p.A. based on the national, an international, and a global index. This chart shows five-year weekly betas for Terna—Rete Elettrica Nazionale S.p.A. from the end of December 2010 to the end of June 2020. Betas are calculated using either the FTSE MIB, the Stoxx Europe 600, or the MSCI World € as the benchmark stock market index. The following descriptive statistics for each beta time series are reported accordingly: mean, median, minimum, maximum, and standard deviation

**Fig. 25:**
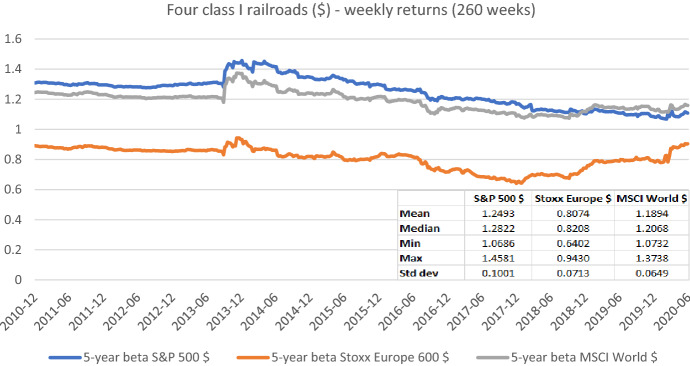
Average betas for the four exchange listed US class I railroads based on the national, a European, and a global index: CSX, Kansas City Southern, Norfolk Southern, and Union Pacific. This chart shows five-year weekly betas for four exchange listed US class I railroads from the end of December 2010 to the end of June 2020. Betas are calculated using either the S&P 500 $, the Stoxx Europe 600 $, or the MSCI World $ as the benchmark stock market index. The following descriptive statistics for each beta time series are reported accordingly: mean, median, minimum, maximum, and standard deviation

## Data Availability

Due to privacy concerns, the authors cannot share the market data obtained from the Refinitiv Datastream database and used to calculate betas of sample firms. The calculations were performed using MS Excel.

## Appendix

See Figs. 18, 19, 20, 21 , 22, 23 , 24 , 25.

## Footnotes

1 Several formulas exist to transform equity betas into asset betas and to “relever”. In these processes, a “debt beta” estimate must be taken into account and leverage must be measured in a meaningful way. If the formulas contain tax rates, it must be decided, whether statutory tax rates or effective tax rates are used. Indepen (2018), in their study commissioned by Ofgem, discuss these problems in their.

2 Using averages of betas based on different window lengths is a procedure that is often applied. For instance, Frontier Economics has applied it since 2008 and still uses it, see Frontier (2021).

3 See “Safe utilities have been more volatile than broader stock market” by Anna Hirtenstein (The Wall Street Journal, June 14, 2020). https://www.wsj.com/articles/safe-utilities-have-been-more-volatile-than-broader-stock-market-11592132401. Accessed 08 March 2023.

4 We also considered *Italgas S.p.A.* as a relevant pure gas network operator. However, as *Italgas S.p.A.* has been publicly listed only since November 7, 2016, we do not include it in our main analyses due to the lack of sufficient data. We do not include *Fluxys Belgium S.A.* – the Belgian gas network operator – in our study due to the very small free float of its stocks (i.e., 90% of its shares are held by *Fluxys S.A.*).

5 Originally, all major indices did not include dividends. Since 1988 the S&P 500 has a version that includes dividends. But in the financial press until today the version without dividends is typically reported. Hartzmark and Solomon (2020) point out, that for most other local indices the situation is similar. They show that this creates distortions in the market that could easily be avoided by reporting the total return versions of the indices in the financial press.

6 For guidance on how to deal with further documented data-issues in the Refinitiv Datastream database, for instance, please refer to the study of Landis and Skouras (2021).

7 Total returns can differ with regard to the assumed reinvestment of corporate payments. While gross total return indices reinvest the relevant payments before tax deductions, net total return indices do this after tax. Hence, for consistency reasons, we make sure that gross total returns are used for all assets examined.

8 Of course, given that the market index itself has no individual non-trading days.

9 The results for other sample firms and data frequencies are similar, thus, we do not report them for brevity.

10 The same also applies to estimations with daily data.

11 Sometimes the use of local indices is defended by the “home” bias of investors. Empirical studies show, that historically many investors preferred to invest at home. Such a bias is fully compatible with an international interpretation of the CAPM or an international CAPM. Belgium, Spain, and Italy have pure electricity operators. So, for diversification reasons, there is no need for Italian investors to hold shares of the Spanish operator. Today the home bias of private investors is also most likely smaller than in the 1990s, when the landmark studies on the home bias appeared. And todays extremely large institutional investors, e.g. CalPERS or the Government Pension fund of Norway, which probably have a large influence on stock prices, at least at the margin, do not seem to have much of a home bias.

12 Theoretically this is not correct. When the CAPM holds internationally, risks that are non-diversifiable in the local market may be diversifiable at the international level, see Grauer et al. (1976). If the national index and the international index are perfectly correlated, using the national index would be theoretically correct, but not if the two indices are less than perfectly correlated.

13 In the Appendix we include similar figures for the other seven firms in our sample.

14 In Fig. 25 in the Appendix, we complement our findings by looking at the average betas of four US Class I railroads. These are calculated using the S&P 500 as a national index and using both international indices, the Stoxx Europe 600 and the MSCI World, denominated in US dollars. The graph shows that betas calculated with the European stock index are substantially lower over the full sample period than betas based on the S&P 500 index and the MSCI World index – which is dominated by US firms. We conclude that since different national and international indices are usually not perfectly correlated, they cannot simply be used interchangeably.

15 See “Elia's German grid buy changes electricity landscape” (Reuters, March 12, 2010). https://www.reuters.com/article/vatten-fall-elia-idUSLDE62B0A220100312. Accessed 08 March 2023.

16 Note that these results only hold for the group of stocks we and Wright et al. (2018) look at, i.e., stocks of firms subject to network regulation. As a consequence of the fact that all betas sum to one—if calculated identically—there must be stocks whose betas increase at low frequencies.

17 Our findings on differences in beta estimates related to different data frequencies are also in line with other academic studies, see e.g. Hawawini (1983).

18 Although Wednesday returns might have the advantage of a slightly lower number of non-trading days.

19 Given a market portfolio with the sum of the weighted stock betas equal to one.

20 We noted in the introduction that because the market-value weighted average of all betas is by definition equal to 1 at all times, it cannot increase in a crisis. An unweighted average of all betas may vary over time.

21 See “Markets crash: How panic spread around the globe” by Julia Kollewe (The Guardian, October, 10 2008). https://www.theguardian.com/business/2008/oct/10/marketturmoil-creditcrunch1. Accessed 08 March 2023.

22 See “U.S. Stocks Sink in Worst Day Since Black Monday: Markets Wrap” by Claire Ballentine, Vildana Hajric, and Sarah Ponczek (Bloomberg, March 11, 2020). https://www.bloomberg.com/news/articles/2020-03-11/asia-stocks-set-for-losses-dow-enters-bear-market-markets-wrap. Accessed 08 March 2023.

23 The most recent data suggest that, on average, betas still seem to remain at the high level even one year after the COVID-19-related shock. We validated this finding by comparing the average asset beta of electricity and gas network operators in our sample with the respective value available in the most recent regulatory publication, which is Frontier (2021). We find that they are very similar.

24 The Vasicek adjustment towards an industry beta, however, requires that it remains relatively constant over time. In practice, this assumption is frequently not satisfied.

25 The five-year betas of *Redes Energeticas* are only available as of the end of June 2012 because the company has been publicly listed since July 9, 2007.
